# Indole-3-acetic acid (IAA) production trait, a useful screening to select endophytic and rhizosphere competent bacteria for rice growth promoting agents

**DOI:** 10.1016/j.mex.2015.02.008

**Published:** 2015-02-20

**Authors:** Hassan Etesami, Hossein Ali Alikhani, Hossein Mirseyed Hosseini

**Affiliations:** Department of Soil Science, College of Agriculture & Natural Resources, University of Tehran, Karaj Iran

**Keywords:** Endophytic, PGP traits, PGPR, IAA, Colonization, Rice

## Abstract

Plants select plant growth promoting rhizobacteria (PGPR) that are competitively fit to occupy compatible niches without causing pathological stress on them. However, when screening bacteria for plant growth promoting (PGP) agents, it is better to select bacteria for achieving the most promising isolates having suitable colonization and PGP traits. In most researches, it has been seen that following incubation, bacterial flora are taken at random from petri dishes for further study. However, this type of selection may remove some superior bacteria in terms of PGP traits and high colonization ability. Therefore, it is essential to study all the isolated bacteria in an economic way and select the best bacteria in terms of PGP traits and high colonization rate. A simple screening method to detect endophytic and rhizosphere bacteria, isolated from the plants in rotation with rice, for rice PGP agents based on a root colonization bioassay and a PGP trait is characterized.

• Selected bacterial isolates based on their IAA producing trait have the potential for more PGP and colonization of rice plant.
• IAA may be the first PGP trait for screening bacteria isolated from plant rotated with rice for rice PGP agents.
• The screening procedure appears to be very effective and less time consuming.

## Graphical abstract

**Figure:**
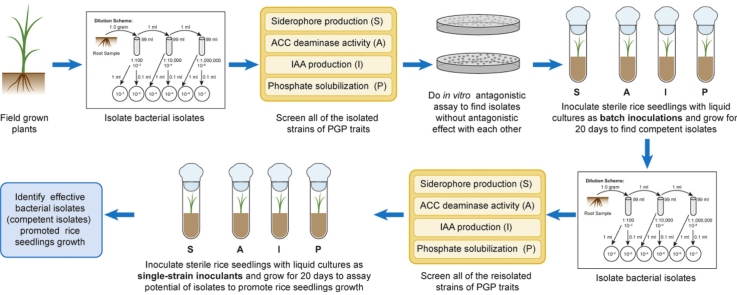

## Method details

1. Collect rhizosphere soil and roots of the healthy plants of interest in rotation with rice, at flowering, randomly from different locations of a field [1].
2. Isolate endophytic and rhizosphere bacterial isolates from these plants according to current methods [2] and store them in a refrigerator at 4 °C for further studies.
  *Note*: Similar bacterial isolates must be grouped based on phenotypic characteristics, as there has not been a possibility of obtaining repeated strains in the collection.
3. Screen all endophytic and rhizosphere strains for plant growth promoting (PGP) traits such as production of Indole-3-acetic acid (IAA) [3], siderophore [4], 1-aminocyclopropane-1-carboxylate (ACC) deaminase [5] and phosphate solubilization [6].
4. Find isolates without any antagonistic effect with each other by an *in vitro* antagonistic assay on all isolates based on the method described by Etesami et al. [1].
  *Note*: This assay must be performed on four groups of plant growth promoting bacteria (PGPR) with different PGP traits namely IAA producing isolates, ACC deaminase producing isolates, siderophore producing isolates and phosphate solubilizing isolates separately to find isolates without any antagonistic effect with each other and having the same PGP traits.
5. Inoculate each of IAA, siderophore and ACC deaminase producing isolates and phosphate solubilizing isolates as mixtures of strains each (isolates without antagonistic effect with each other and having the same PGP trait as batch inoculations) on rice plant seedlings in sterile Hoagland’s medium containing 8 mM (NH_4_)_2_SO_4_ under *in vitro* conditions as described by Etesami et al. [1] to find competent endophytic and rhizosphere isolates and grow for 20 days.
  *Note* 1: Before assaying colonization to find competent endophytes, establish separate tests that seeds of rice cultivar do not harbor any endophytic bacteria.
  *Note* 2: Aseptic technique must be used throughout and the surface-sterilization procedure must be effective to verify that the same bacteria inoculated to sterile rice seedlings could be re-isolated from these seedlings (to fulfill Koch’s postulate), and examine their endophytic competence (infection and persistence characteristics).
6. Isolate endophytic and rhizosplane bacterial isolates inoculated on rice seedlings after 20 days.
7. Screen all endophytic and rhizoplane strains isolated from rice seedlings for the same plant growth promoting (PGP) traits as mentioned above.
  *Note*: this step was performed for the first time to determine whether bacteria reisolated from rice seedlings can have other PGP traits. For example, whether ACC deaminase producing isolates can also produce IAA or siderophore or solubilize phosphate. In our studies [1], [7], it was recognized that all of the reisolated strains from rice seedlings were able to produce IAA. In other words, each isolate having PGP trait had at least the ability of production of IAA too. Therefore, this step is not essential for doing a new assay to achieve the most promising isolates in colonization and promotion of rice plant growth.
8. Inoculate each of IAA, siderophore and ACC deaminase producing isolates and phosphate solubilizing isolates as single-strain inoculants on rice plant seedlings in sterile Hoagland’s medium containing 8 mM (NH_4_)_2_SO_4_ under *in vitro* conditions as described by Etesami et al. [1] to find competent endophytic and rhizosphere isolates and grow for 20 days to assay potential of the isolates to promote rice seedlings growth.
9. Identify the best isolate in terms of high colonization and promotion of rice seedlings growth

## Method validation

**Fig. 1:**
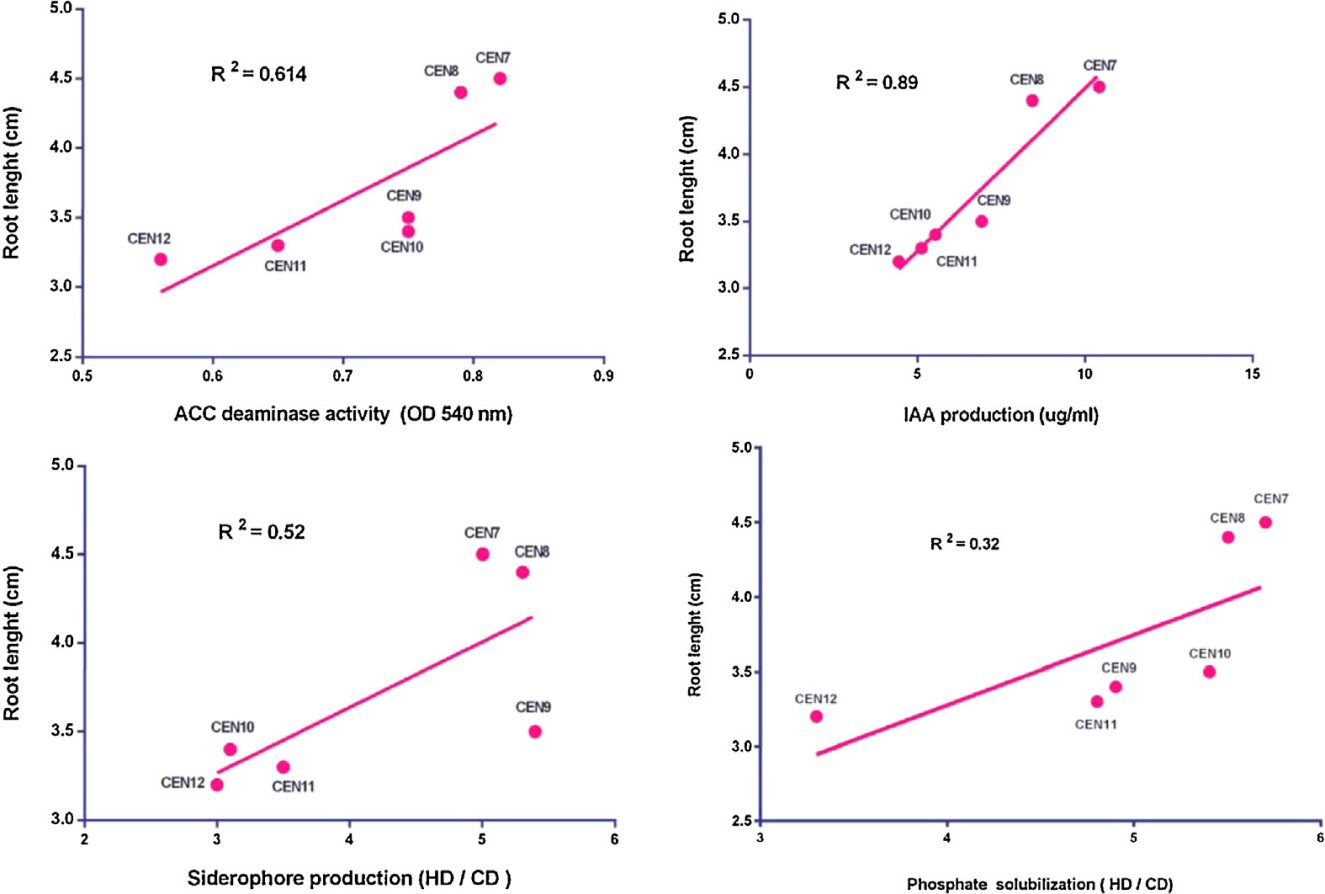
Linear regression showing the relationship among ACC deaminase activity, IAA production, siderophore production, and phosphate solubilization of bacterial strains and their effect on root elongation of rice seedlings 20 days after inoculation [1].

To confirming the efficiency of the bioassay, ten isolates isolated from berseem clover or canola plants grown at field, which had been screened only based on the production of IAA, were retested, using the same procedures. As additional negative controls, two IAA non-producing isolates were also included. Screening to detect isolates with good potential as rice growth-promoting agents indicated that seven out of ten endophytes, inoculated on five rice cultivars (*Hashemi*, *Khazar*, *Gohar*, *Kadus*, *Sadra*), were able to colonize within roots and promote plant growth. According to the results obtained, 7 out of 10 isolates tested (70%) behaved as potentially good PGP and colonizing agents. Seedlings inoculated with IAA producing isolates yielded more shoot biomass (stem plus leaves), root length and colonization than the control plants inoculated with IAA non-producing strains and plants inoculated with PGPR producing other PGP traits [1], [8]. These assays were performed twice on rice cultivars. Based on this bioassay method, a significant relationship among IAA and ACC deaminase production traits, and root length than other PGP traits (siderophore production and phosphate solubilization) was observed (Fig. 1) [1].

**Fig. 2:**
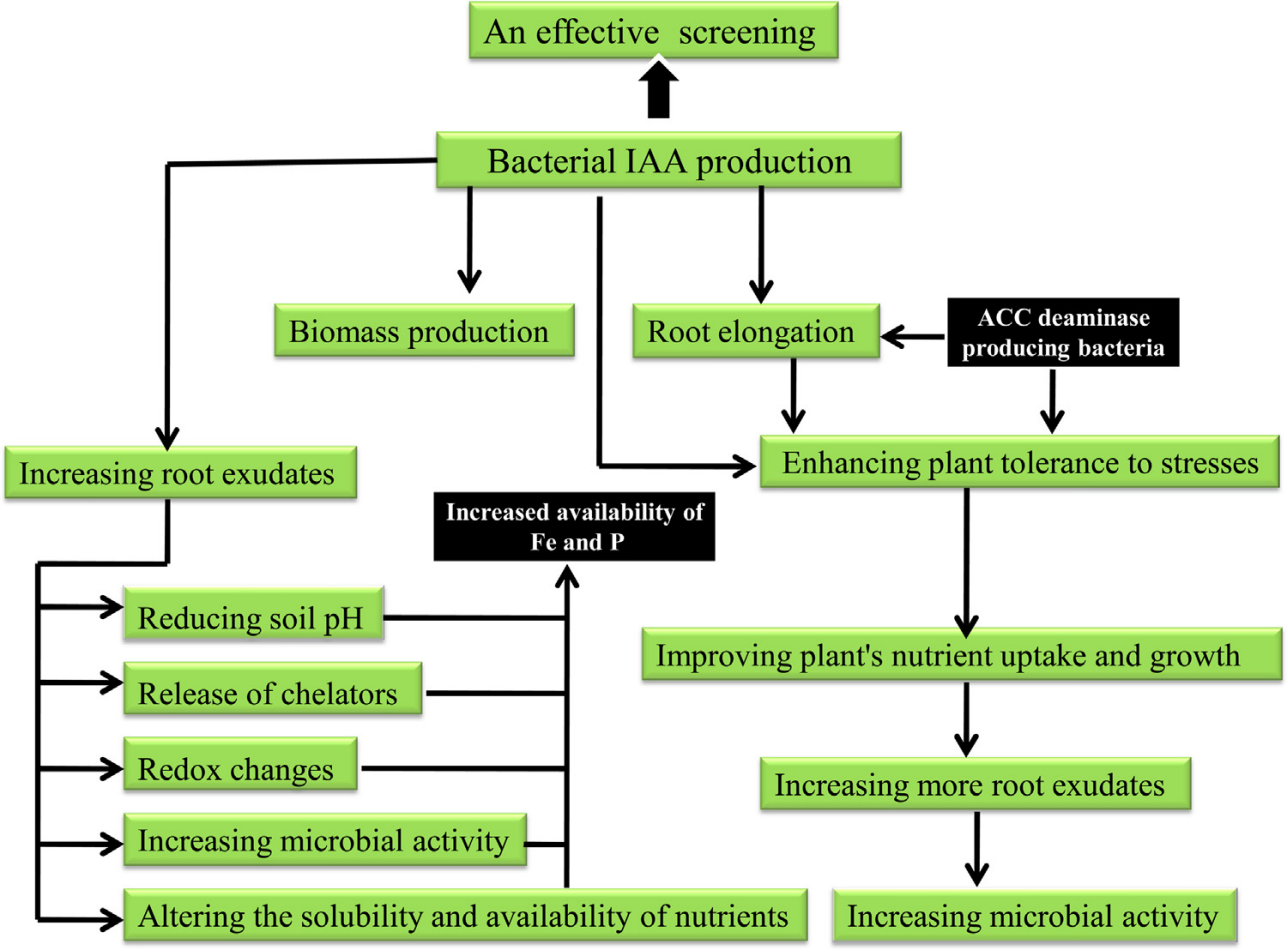
Functions of bacterial IAA in obviating some of the roles of ACC deaminase and siderophore producing bacteria and phosphate solubilizing bacteria.

There are many evidences that IAA may be the first PGP trait compared to ACC deaminase activity, siderophore production and phosphate solubilization traits for screening rhizosphere and endophytic bacteria for rice plant PGP agents below:

• Bacterial IAA contributes to circumvent the host defense by derepressing the IAA signaling in the plant; IAA also can have a direct effect on bacterial survival and its resistance to plant defense [9].
• The success of invasion and survival within the host also requires that bacteria overcome plant defense responses triggered after microbial recognition, a process in which surface polysaccharides, antioxidant systems, ethylene biosynthesis inhibitors and virulence genes are involved [10]. It can be speculated that IAA production trait is part of the strategy used by IAA producing bacteria to circumvent the plant defense system [9].
• IAA is a plant hormone with no apparent function in bacterial cells, and it could be speculated that IAA production may improve the fitness of the plant–bacterium interaction [11].
• Since the first step of bacteria invasion in plant root comprises of the attachment of isolates onto epidermal cells of the root surface, where root hair zone shows one of the major sites of primary colonization (mainly on the basal region of emerging hairs), it is possible that IAA producing bacteria by increased root system can colonize plant roots better than other bacteria. In addition, IAA levels weaken plant defence mechanisms making colonization easier [12], [13], [14], [15].
• Bacterial IAA can loosen plant cell walls and as a result promotes an increasing amount of root exudation that provides additional nutrients to support the growth of rhizosphere bacteria [12], [13]. Since endophytic microbial communities originate from the soil and rhizosphere [16], [17], bacterial IAA can attract more rhizoshere bacteria by increasing more amount of root exudation. Since bacterial IAA stimulates the development of the root system of the host plant [18], IAA producing isolates can improve the fitness of plant-microbe interactions [18], [19].
• It is known that bacterial IAA can loosen plant cell walls and as a result promotes an increasing amount of root exudation that provides additional nutrients to support the growth of rhizosphere bacteria [12], [13].
• IAA stimulates overproduction of root hairs and lateral roots in plants and release of saccharides from plant cell walls during the elongation [20]. Saccharides are a source of nutrients for microorganisms and can increase the colonization ability of plant-associated bacteria [19].
• Bacterial IAA increase root surface area and length, and thereby provides the plant greater access to soil nutrients and water uptake [21].
• In view of function of bacterial IAA in increased root system, it is may proposed that IAA producing bacteria can provides more number of active sites and access to colonization for other PGPRs. For example, the presence of PGPRs in the root vicinity could improve ability of rhizobia to compete with indigenous populations for nodulation [22].
• It is hypothesized that the secretion of IAA may modify the microhabitat of epiphytic bacteria by increasing nutrient leakage from plant cells; enhanced nutrient availability may better enable IAA-producing bacteria to colonize the phyllosphere and may contribute to their epiphytic fitness [19].
• Bacterial IAA can obviate to a certain extent the function of ACC deaminase and siderophore producing bacteria and phosphate solubilizing bacteria (Fig. 2)

It may be suggested that plants select endophytic and rhizosphere bacteria with these traits or that these bacteria harbor other traits that allow them to more effectively reach and establish themselves in rhizoplane and the inner plant tissue [23]. Therefore, screening of the rhizosphere and endophytic bacteria for their *in vitro* potential of IAA production could provide a reliable base for selection of effective PGP bacteria. Many studies have shown that the interaction of IAA-producing bacteria with plants might posit that since IAA activates the transcription of ACC synthease, these bacteria should all ultimately result in the production of relatively high concentrations of ACC and subsequently inhibitory levels of ethylene. Thus, in the absence of some other mechanism, IAA-producing bacteria might all be expected to ultimately be inhibitory to plant growth. However, according to Glick [24] this is in fact not the case because as plant ethylene levels increase, the ethylene that is produced feedback inhibits IAA signal transduction thereby limiting the extent that IAA can activate ACC synthase transcription.

**Fig. 3:**
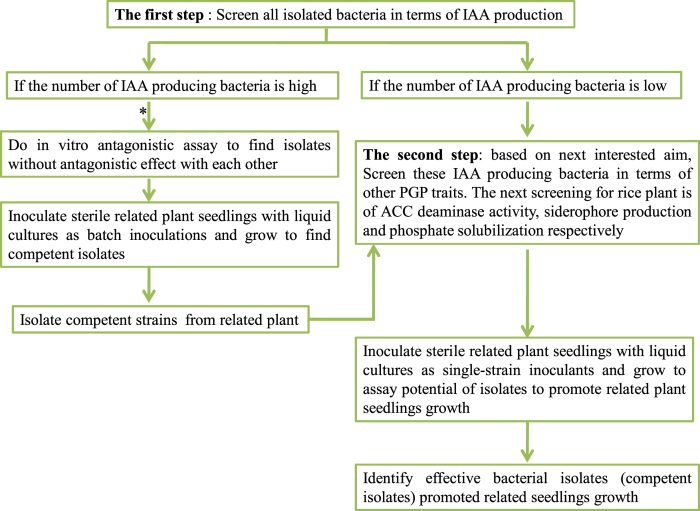
Steps of doing the screen of rhizophere and endophytic bacteria isolated from a crop in rotation with rice. *: these steps are performed to reduce the number of IAA producing bacteria and find the best competent endophytic and rhizosphere isolates for further studies.

Therefore, the screening procedure appears to be very effective and less time consuming (Fig. 3). This screening procedure can be used for any crop in rotation with rice, as we studied this assay for clover and canola plants rotated with rice in Iran.

## Additional information

PGPRs may use different mechanisms to enhance plant growth as experimental evidence suggests that the plant growth stimulation is the net result of multiple mechanisms that may be activated simultaneously [25]. Despite their different mechanisms of action, their use has not been developed to its full potential due to inconsistencies in their performance, and their commercialization has been limited to a few developed countries. In many cases, PGPRs fail to induce the desired effects when applied in the field. This might be due to insufficient rhizosphere and/or plant colonization, which is as an important step required for exhibiting beneficial effects [26]. Many studies have been performed on colonization of bacteria on plants genotypically but not phenotypically. Several methods have been used to demonstrate that root colonization is taking place, including use of fluorescence techniques, antibiotic-resistant mutants, and marker genes, such as LUX and GUS. However, these methods are relatively expensive and time consuming [27], [28]. Based on this method, isolated bacteria will be the best in colonization and promotion of rice plant growth. Since the final aim after selecting the best isolate will be to introduce these isolates as a biofertilizer (suitable for pudding) for farmers, to find how much of chemical fertilizers (maximum yield) those bacteria can replace, we should select bacteria that have been isolated in the presence of N. We inoculated rice plant with both endophytic isolates and rhizosphere isolates isolated from the plant roots. Because microfloral populations already resident within the host plant may well influence and be influenced by rhizosphere bacteria [29]. In addition, several studies have reported that endophytic microbial communities originate from the soil and rhizosphere [16], [17], [30]. We inoculated these isolates as mixtures of strains on rice seedlings, because it should be realized this competition mimics the situation in raw soil, which contains approximately 10^8^ bacteria g^1^ [31]. Based on our studies, IAA may be the first PGP trait for screening bacteria isolated from plants in rotation with rice PGP agents. The next screen of IAA producing isolates for rice plant promoting agents may be of ACC deaminase activity, siderophore production and phosphate solubilization respectively. Since Fe, NH_4_^+^ and phosphorus were available to rice seedlings in sterile Hoagland’s medium and the same in rice fields due to anaerobic conditions (high availability of Fe ^+2^ and phosphorus), it is seemed rice seedlings did not have more need to attract isolates helping the increase of solubility of these nutrients. However, due to possibility of production of ethylene under constant flooding conditions, ACC deaminase producing isolates could be next option to be attracted by rice seedlings.
